# Role of Comorbidities in Optimizing Decision-Making for Allogeneic Hematopoietic Cell Transplantation

**DOI:** 10.4084/MJHID.2010.015

**Published:** 2010-06-09

**Authors:** Mohamed L. Sorror, Rainer F. Storb

**Affiliations:** 1 Fred Hutchinson Cancer Research Center; 2 University of Washington School of Medicine, Seattle, WA

## Abstract

Allogeneic conventional hematopoietic cell transplantation (HCT) following high-dose, myeloablative conditioning regimens has been used since the 1970’s as potentially curative treatment for patients with malignant, hematological disorders. The toxicities of conditioning regimens have limited conventional HCT to relatively young patients in otherwise good medical condition. With the development of less toxic nonmyeloablative regimens and improvements in supportive care, increasing numbers of older and medically infirm patients have been treated by allogeneic HCT. Until recently, there has been almost no effort to evaluate the prevalence of comorbidities among HCT recipients and their impact on outcomes. We first evaluated the Charlson Comorbidity Index (CCI) developed for patients with solid malignancies, for this purpose. While useful, it lacked sensitivity and specificity for the HCT setting. We next introduced the HCT-specific comorbidity index (HCT-CI) which was based on objective laboratory data to better define comorbidities. Here, we describe this development and illustrate the usefulness of the HCT-CI in predicting HCT outcomes in patients with myeloid and lymphoid malignancies undergoing allogeneic transplantation.

## Introduction:

Allogeneic conventional HCT is considered potentially curative for patients with malignant or non-malignant hematological diseases. Conditioning regimens for conventional HCT have been intensified to the limits of organ tolerance in order to optimize disease eradication. Consequently, serious toxicities to organs, such as gut, lung, kidney, heart, and liver have been observed which, additionally, have limited the ability to deliver adequate doses of postgrafting immunosuppression needed for control of GVHD. Until recently, these regimen-related toxicities associated with myeloablative conditioning have limited allogeneic HCT to patients without significant co-morbidities who were less than 55 to 60 years old. This age restriction has been unfortunate since the median ages of patients with most candidate diseases for HCT, e.g., acute and chronic leukemias, myelodysplasia (MDS), multiple myeloma, and lymphomas, have ranged from 65 to 70 years.

In an effort to expand treatment options for patients with hematological malignancies and based on results from a series of canine studies,^1^–^4^ a truly nonmyeloablative regimen of 2 Gy TBI with or without fludarabine, 90 mg/m^2^, has been introduced to older and medically infirm patients before allogeneic HCT from related or unrelated donors.^5^,^6^ The conditioning regimen’s major role has been host immunosuppression. Effective postgrafting immunosuppression with MMF and CSP has been crucial in this approach with the aim of both enhancing hematopoietic engraftment and controlling GVHD. There has been little direct antitumor effect from the conditioning regimen. Instead, the approach has relied predominantly on the generation of donor T cell (and/or NK cell)-mediated graft-versus-tumor effects for eradication of cancer. The use of this nonmyeloablative regimen has expanded the use of HCT to include elderly and medically infirm patients with various hematological disorders.^7^–^9^

Age has been frequently cited as an important prognostic variable in HCT. Historical age cutoffs have been 55 and 60 years, respectively, largely influenced by the type of HCT donor (related versus unrelated). The reason for the age cutoffs has been prohibitive regimen-related toxicity and mortality in older patients. It has also been suggested that older patients were at higher risk of GVHD resulting in worse survivals. Most reports on age and HCT outcomes, however, have ignored comorbidities, which might have been confounding factors. Several investigators have studied single organ comorbidities in the context of predicting same organ toxicity after HCT. Comprehensive assessment of the interaction between multiple comorbidities and their impacts on HCT outcomes has become increasingly important given both increasing age of the Western population along with increasing prevalence of cancer and comorbidities^10^ and the increasing enrollment of patients aged >60 years in HCT clinical trials.^11^

## Comorbidities using the Charlson Comorbidity Index (CCI):

In the field of cancer, investigators have found variable interactions between a given primary disease and different comorbidities based on type and severity of organ involvements. As a result, several indices have been created to rate the impacts of different comorbidities on the primary disease. The Charlson Comorbidity Index (CCI)^12^ included 19 comorbidities which have been selected and weighted based on their strength of associations with mortality. The CCI has been the most widely used comorbidity index to predict mortality risks in various solid malignancies^13^–^21^.

We used the CCI in a retrospective study to compare pretransplant comorbidity differences among recipients of nonmyeloablative (n=60) and myeloablative HCT (n=72) from unrelated donors.^22^ At the time of HCT, nonmyeloablative patients had more often high-risk diseases (*P*=0.02); were older (median age, 54 versus 41 years, *P*<0.0001); had more preceding chemotherapy regimens (3 versus 1, *P*=0.01); had more frequently failed myeloablative HCT (*P*<0.0001); and received more often peripheral blood stem cell grafts (*P*<0.0001) than myeloablative patients. In addition, nonmyeloablative patients had higher CCI scores compared to myeloablative patients (scores of 1–2 and ≥ 3, 35% and 18% compared to 12% and 0%, respectively, *P*<0.0001) at the time of HCT.

After HCT, nonmyeloablative patients experienced less gastrointestinal (*P*<0.0001), hepatic (*P*=0.02), hemorrhagic (*P=*0.005), infectious (*P=*0.09), and metabolic (*P=*0.03) grades III–IV toxicities. Further, there were trends for less neurological, renal, and pulmonary grades III–IV toxicities (*P=*0.1 for each). In particular, nonmyeloablative patients had less (32% versus 69%, *P*<0.0001) overall grade IV (life-threatening) toxicities than myeloablative patients. No single cases of veno-occlusive disease or mucositis was detected among nonmyeloablative compared to 18% and 72% among myeloablative patients, respectively. Also, nonmyeloablative patients experienced less grades III–IV acute GVHD (*P*=0.03). The lessened cumulative incidences of day 100 (12% versus 18%, *P*=1.4) and 1-year (20% versus 32%, *P*=1.4) NRM among nonmyeloablative patients did not reach statistical significance. After adjustment for pretransplant differences, including comorbidity scores, statistically suggestive or significant lower hazard ratios (HR) for day 100 (0.2, *P=*0.07) and 1-year (0.3, *P=*0.04) NRM were found for nonmyeloablative patients, confirming the importance of a single scoring system for comorbidities. In multivariate analyses of risk factors for outcomes, comorbidities as scored by the CCI, proved to be the only independent factor for predicting overall grade IV toxicity (HR were 2.9 and 5.5 for scores 1–2 and ≥ 3, respectively, *p=*0.06) and NRM (HR were 2.4 and 10.5, respectively, *p=*0.04). Cumulative incidence and Kaplan Maier curves showed linear increases in overall grade IV toxicities, NRM, and worsening survival with increasing CCI scores, whereby better outcomes were observed among nonmyeloablative compared to myeloablative patients with similar CCI scores. In a concurrent study, the CCI was important in predicting NRM among recipients of HLA-matched related HCT.^23^

## An HCT-specific comorbidity index (HCT-CI):

The CCI lacked sensitivity in detecting several comorbidities among HCT recipients, given that scores >0 were detected among only 35% of all HCT patients (12% among myeloablative patients).^22^ This was thought to be due to not well-defined definitions of some comorbidities, such as hepatic and pulmonary. In addition, relatively frequent comorbidities among HCT patients, such as infections, were not included in the CCI.

In order to improve sensitivity, a study was designed which included 1055 consecutive recipients of allogeneic HCT between 1998 and 2004 who had various hematological diseases, and of whom 249 received nonmyeloablative and 761 myeloablative conditioning. Patients were randomly assigned to training (n=708) and validation (n=347) sets.^24^ Novel definitions were modeled for hepatic and renal comorbidities by using actual laboratory data and for pulmonary and cardiac comorbidities by using test results of organ function. Also, new integer weights of comorbidities were calculated based on HRs from Cox proportional hazard models of 2-year NRM, which were adjusted for disease risk, age, and conditioning regimen intensity. The new HCT-CI consisted of 17 comorbidities including three comorbidities that were not represented in the CCI, obesity, peritransplant infections, and psychiatric disturbances. HCT-CI scores of 0, 1, 2, 3, and ≥ 4 predicted 2-year NRM of 9%, 14%, 27%, 41%, and 43%, respectively, among patients of the training set.

**Figure 1:**
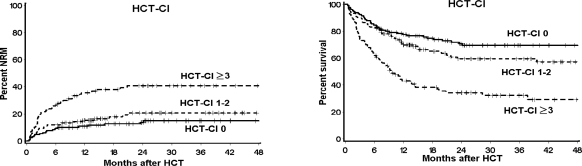
NRM and survival by HCT-CI scores among patients of the validation set.^24^ Patients with HCT-CI scores of 0, 1–2, and ≥ 3 had cumulative incidences of NRM of 14%, 21%, and 41% and survival rates of 71%, 60%, and 34%, respectively. This research was originally published in Blood. Sorror ML, Maris MB, Storb R, Baron F, Sandmaier BM, Maloney DG, Storer B. Hematopoietic cell transplantation (HCT)-specific comorbidity index: a new tool for risk assessment before allogeneic HCT. Blood. 2005;106(8):2912–9. © the American Society of Hematology.

When applied to data from the validation set, HCT-CI scores of 1–2 and ≥ 3 were found in 34% and 28% of patients compared to CCI scores of 1 and ≥ 2 in only 10% and 3% of patients, respectively. Most importantly, HCT-CI scores of 0, 1–2, and ≥ 3 showed linear predictions of NRM (14%, 21%, and 41%) and survival (71%, 60%, and 34%), respectively (**Figure 1**). In addition, HCT-CI scores had higher discriminative power than CCI scores both for NRM (*c* statistic of 0.692 versus 0.546, *P* < 0.001) and survival (*c* statistic of 0.661 versus 0.561, *P* < 0.001).

## HCT-CI and outcomes after conditioning regimens of different intensities:

### Patients with acute myeloid leukemia (AML) or myelodysplasia (MDS)

We compared outcomes among patients with AML (n=391) or MDS (n=186) given either nonmyeloablative (n=125) or myeloablative HCT (n=452).^25^ The median age of nonmyeloablative patients was 60 years compared to 46 years among myeloablative patients. In an initial analysis of outcomes among all patients, high HCT-CI scores and high disease risk independently predicted non-relapse mortality (NRM, *p*<0.0001 and *p*=0.004), overall survival (OS, *p*<0.0001 and *p*<0.0001), and relapse-free survival (RFS, *p*<0.0001 and *p*<0.0001), respectively. This allowed us to divide patients into four risk groups based both on comorbidities and disease risks (**Table 1**).

Cumulative incidences of NRM tended to be lower and relapse rates higher among nonmyeloablative compared to myeloablative patients resulting in comparable rates of OS and RFS across all risk groups, even though nonmyeloablative patients were older than those given myeloablative conditioning. Novel anti-tumor agents combined with nonmyeloablative HCT should be explored among patients with high comorbidity scores and advanced disease.^25^

### Patients with lymphoma or chronic lymphocytic leukemia (CLL)

Myeloablative allogeneic HCT has been associated with high regimen-related mortality (up to 60%) among patients with lymphoma or CLL.^26^–^29^ In order to get around this problem, nonmyeloablative conditioning regimens have been explored. A recent analysis compared outcomes among 152 older (median age, 60 years) patients given nonmyeloablative conditioning to those among 68 younger (median age, 46 years) patients given myeloablative conditioning, stratifying for the HCT-CI.^30^

**Figure 2:**
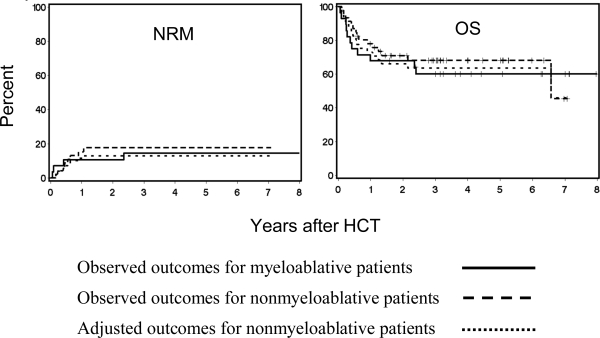
Cumulative incidence estimates of NRM and Kaplan Meier survival estimates among nonmyeloablative compared to myeloablative patients with lymphoma or CLL and HCT-CI score 0.^30^ NRM (18% versus 15%; respectively, P = 0.74) and OS (68% versus 60%; respectively; P = 0.75) were comparable among nonmyeloablative patients with HCT-CI scores of 0 compared to myeloablative patients. Differences remained statistically not significant (HR: 0.90; P = 0.91 and HR: 1.94; P = 0.27, respectively) after adjustment for other risk factors. This research was originally published in Blood. Sorror ML, Storer BE, Maloney DG, Sandmaier BM, Martin PJ, Storb R. Outcomes after allogeneic hematopoietic cell transplantation with nonmyeloablative or myeloablative conditioning regimens for treatment of lymphoma and chronic lymphocytic leukemia. Blood. 2008; 111(1):446–52. © the American Society of Hematology.

**Figure 3:**
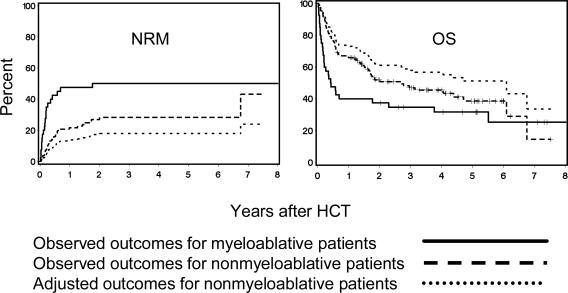
Cumulative incidence estimates of NRM and Kaplan Meier survival estimates among nonmyeloablative compared to myeloablative patients with lymphoma or CLL and HCT-CI score ≥1.^30^ NRM was statistically significantly lessened (28% versus 50%; respectively, P = 0.009) and OS rates were more favorable (47% versus 35%; respectively; P = 0.04) among nonmyeloablative patients with HCT-CI scores of ≥1 compared to myeloablative patients. Further, differences became more significant for NRM (HR: 0.19; P < 0.001) and OS (HR: 0.33; P = 0.007) after adjustment for other risk factors. This research was originally published in Blood. Sorror ML, Storer BE, Maloney DG, Sandmaier BM, Martin PJ, Storb R. Outcomes after allogeneic hematopoietic cell transplantation with nonmyeloablative or myeloablative conditioning regimens for treatment of lymphoma and chronic lymphocytic leukemia. Blood. 2008; 111(1):446–52. © the American Society of Hematology.

We found that patients without comorbidities both in the nonmyeloablative and myeloablative cohorts had comparable NRM, OS, and progression-free survivals (**Figure 2**). However, nonmyeloablative patients with comorbidities had lower NRM (*p* = 0.009) and better OS (*p* = 0.04) than myeloablative patients (**Figure 3**). These differences became more significant after adjusting for other variables; also adjusted progression-free survival was better (*p* = 0.01). This suggests that younger patients with comorbidities would benefit from reducing conditioning intensity.

## Conclusions:

The HCT-CI provided simple and reliable scoring of pre-transplant comorbidities that predicted NRM and survival. The index still needs validation among larger patient samples in multi-center settings. Comorbidity data used in the index will likely become as important as defining cancer diagnosis, disease stage and other, more familiar prognostic variables.^31^

## Figures and Tables

**Table 1:** Two-year NRM, relapse, OS, and RFS incidences among 4 risk groups of nonmyeloablative and myeloablative patients with AML or MDS. Donors were either related (n=301) or unrelated (n=276).^25^

**Risk groups**	**Patients**	**NRM (%)**	**Relapse (%)**	**OS (%)**	**RFS (%)**
Group I (HCT-CI scores 0–2 and low-risk diseases)	Myeloablative (n=138)	11	14	78	75
	Nonmyeloablative (n=28)	4	33	70	63
Group II (HCT-CI scores 0–2 and intermediate and high-risk diseases)	Myeloablative (n=176)	24	34	51	43
	Nonmyeloablative (n=34)	3	42	57	56
Group III (HCT-CI scores ≥ 3 and low-risk diseases)	Myeloablative (n=52)	32	27	45	41
	Nonmyeloablative (n=19)	27	37	41	36
Group IV (HCT-CI scores ≥ 3 and intermediate and high-risk diseases)	Myeloablative (n=86)	46	34	24	20
	Nonmyeloablative (n=44)	29	49	29	23

Reprinted with permission. © 2008 American Society of Clinical Oncology. Sorror, M. L. et al. J Clin Oncol; 26:4912–4920 2008.
